# Gambling and the COVID-19 pandemic in the province of Quebec (Canada): results from an online cross-sectional survey of people who had gambled within the last 12 months

**DOI:** 10.1136/bmjopen-2024-097944

**Published:** 2026-03-26

**Authors:** Magaly Brodeur, Marie-Ève Fortier, Nathalie Carrier, Sophie Audette-Chapdelaine, Anne-Marie Auger, Annie-Claude Savard, Sylvia Kairouz

**Affiliations:** 1 Department of Family and Emergency Medicine, University of Sherbrooke, Sherbrooke, Quebec, Canada; 2 Department of Psychology, University of Sherbrooke, Sherbrooke, Quebec, Canada; 3 Sherbrooke University Hospital Research Centre, Sherbrooke, Quebec, Canada; 4 School of Social Work and Criminology, Laval University, Québec City, Quebec, Canada; 5 Department of Sociology and Anthropology, Concordia University, Montreal, Quebec, Canada

**Keywords:** COVID-19, MENTAL HEALTH, Behavior, Impulse control disorders, PUBLIC HEALTH

## Abstract

**Abstract:**

**Objectives:**

This article presents the quantitative phase of a two-phase mixed methods study. The main objective of this article is to examine how the COVID-19 pandemic affected adult gamblers’ gambling behaviours and mental health in Quebec.

**Design:**

A cross-sectional online survey was used to collect data.

**Setting:**

Quebec (Canada).

**Participants:**

A sample of 973 gamblers completed the problem gambling severity index (PGSI). The participants were French-speaking adults living in the province of Quebec, and they had gambled at least once in the preceding 12 months.

**Main outcome measures:**

Descriptive analysis, *χ*^2^ or the Monte Carlo estimation, Kruskal–Wallis and multivariate logistic regression analyses were conducted.

**Results:**

In the sample, 24.7% were no-risk gamblers, 18.6% were low-risk gamblers, 27.9% were moderate-risk gamblers and 28.9% were high-risk or problem gamblers. Most of the participants reported an increase in their online gambling, in the duration in which they were available for gambling, and the frequency with which they gambled during the pandemic. The results of this study suggest positive associations between PGSI scores and symptoms of depression and anxiety. In this study, 11 independent variables explained 50.9% of the variance of problematic gambling (ie, PGSI ≥ 3) during the COVID-19 pandemic. These variables are related to types of gambling, psychosocial factors, changes in tobacco use and gambling expenditures and high gambling frequency in the last 12 months.

**Conclusion:**

Given the general increase in gamblers’ various gambling behaviours during the pandemic, along with the observed impacts on their mental health and reluctance to seek assistance for problematic gambling, future research must explore the mental health of gamblers after COVID-19-related public health measures were eased.

**STRENGTHS AND LIMITATIONS OF THIS STUDY**

- This study presents an overview of the impacts of the COVID-19 pandemic on 973 gamblers in Quebec.
- The sample of this study is composed of a broad range of problem gambling severity index scores that could help distinguish the impacts of the pandemic on low-risk, moderate-risk and high-risk/problem gamblers.
- Given the proportion of high-risk/problem gamblers in the sample, an overview of the factors that put individuals at risk for problematic gambling during the pandemic is provided in this study.
- Given the non-random sampling used in this study, the gambling behaviours of the participants may not be representative of the general population.
- Although the use of short versions of the mental health scales may limit the interpretation of participants’ mental states, the study provides an overview of the impacts of the COVID-19 pandemic on the participants’ mental states.

## Introduction

 Gambling is recognised as an important public health issue in many countries.^1^,^8^ The prevalence of problem gambling is estimated to be between 0.12% and 5.80% across countries.^3^ Psychiatric comorbidities, such as mood disorders, substance abuse disorders and anxiety, are common among people involved in high-risk gambling.^9 10^ Numerous other far-reaching harmful effects are also associated with excessive gambling practices (e.g., loss of employment or financial instability, increased conflicts and relationship difficulties, increased psychological distress, suicide, etc.) and they can affect families and communities.^45 11^,^20^

The COVID-19 pandemic has had a major impact on the lives of individuals, communities, health systems, industries and economies worldwide. The lifestyle of populations around the world has been directly affected by COVID-19-related public health measures and restrictions, such as lockdowns, physical distancing, prohibited gatherings, adaptation to telework and sudden industry shutdowns.^21^,^26^ Early in the pandemic, many stakeholders were concerned about the impact that the COVID-19 pandemic could have on those who gamble. Previous research observed that individuals who found themselves in situations of social isolation and emotional stress increased their use of alcohol and drugs to cope with sanitary crises.^27^ An increase in gambling has also been observed during periods of crisis.^28^,^30^ Moreover, financial concerns, social isolation, anxiety and boredom generally increased during the pandemic, and they have been thought to have impacted gambling behaviours.^31^,^33^ Furthermore, some concerns existed, that is, the imposed public health measures related to the pandemic would lead to an increase in online gambling,^34^,^36^ which was associated with an increased risk of gambling-related harms and problems, given its unlimited access and anonymity.^37^,^41^ Previous studies suggest that online gambling is associated with problem gambling rates ranging between 5% and 40%.^42 43^

The gambling industry and gamblers were directly affected by the pandemic and the unexpected shutdown of gambling facilities around the world.^3444^,^48^ In the province of Quebec (Canada), the gambling context was deeply affected by the COVID-19 pandemic. As of mid-March 2020, Loto-Québec, the state-owned company that has the mandate to conduct and manage gambling in the province, closed all casinos, bingo halls, video lottery terminals and horse racing venues. They also suspended the sale of lottery tickets in land-based retail for a few weeks.^49^,^51^

This study is part of a larger project that seeks to identify the impacts of the pandemic on gamblers and explore the experiences of gamblers during the pandemic in the province of Quebec. For this project, a two-phase mixed methods study was conducted.^52^ The first phase used a cross-sectional survey to collect quantitative data. The second phase used semi-structured interviews to collect qualitative data. This article presents the quantitative phase that had the following three specific objectives: (1) Describe the gambling habits and gambling problems of gamblers, (2) Explore the general impacts of the pandemic on the mental health and gambling habits of gamblers and (3) Identify factors that predisposed people to high-risk gambling during the pandemic.

## Methods

Using a cross-sectional design, French-speaking adults living in Quebec were surveyed, who had gambled at least once in the preceding 12 months. Participants were recruited via an invitation to complete an online questionnaire that was posted on social media sites and on the websites of different organisations (Loto-Quebec, the helpline *Gambling: Help and Referral*). The link to the online survey was also sent in emails to the researchers, inviting them to share it with their network. The questionnaire was made accessible between February 16 2021, and March 15, 2021.

The 85-item questionnaire was divided into four sections.^1^ The first section pertained to demographic characteristics and allowed participants to indicate a change that may have taken place during the pandemic (e.g., marital and employment status). The second section addressed the participants’ perception of the general impacts COVID-19 and public health had on them (social relationships, family life, lifestyle habits and substance use). It included items inspired by a Statistics Canada survey related to the impacts of COVID-19^53^ and validated instruments to assess participants’ mental health, such as anxiety and depression. The patient health questionnaire-4 (PHQ-4) is a validated screening tool comprising two subscales: the generalised anxiety disorder-2 (GAD-2), to screen for anxiety symptoms and the patient health questionnaire-2 (PHQ-2), to screen for depressive symptoms.^54^,^56^

The items in the third section were designed to examine gamblers’ gambling habits, the impact COVID-19 had on their gambling behaviours (frequency of gambling, length of gambling sessions, amount of money spent on gambling, etc.), their self-assessment regarding the presence of problem gambling in the present or the past, and the use of self-exclusion on Loto-Quebec. It also included the problem gambling severity index (PGSI) to assess problem gambling. The PGSI is a nine-item, validated instrument designed to measure risk of problem gambling in the general population. The PGSI total score is used to classify gambling in the following four levels: no-risk gambler (0), low-risk gambler (1–2), moderate-risk gambler (3–7) and high-risk gambler (8 and over).^57 58^

In this article, the term “problem gambler” is used to refer to the last level of the PGSI, which can also be considered a high-risk gambler. However, for the regression analysis in this study, moderate-risk gamblers (PGSI 3–7) and problem gamblers (PGSI ≥ 8) were considered together using the term “problematic gambling” (PGSI ≥ 3) to define them. This aligned with a national survey created to evaluate French gambling practices.^59^ However, the researchers of this study are aware that moderate-risk gamblers and problem gamblers are heterogeneous, and they frequently differ on various variables.^60 61^

The fourth section of our questionnaire was designed to collect general information about participants’ physical and mental health status and experiences with the healthcare and social services system. A detailed description of the questionnaire is provided in the protocol of our study.^52^

This study initially included 1654 participants. However, 101 participants were excluded due to various reasons, including refusal to consent (n = 11), incomplete demographic information (n = 42), being a minor (n = 2), residing outside Quebec (n = 5) and no participation in gambling in the past 12 months (n = 41). In addition, 578 participants did not complete the PGSI questions and were subsequently excluded. After analysis, two more participants were excluded, as, according to their answers, they had not gambled in the last 12 months. Therefore, the final sample size for analysis comprised 973 participants.

### Outcomes

The regression model tested included 21 independent variables to see which would predict problematic gambling (PGSI ≥ 3): the impact of the COVID-19 pandemic on marital status, job loss, household income, consumption of alcohol, tobacco, cannabis, drug and pornography, gambling on the lotto, in casinos, on slot machines, on electronic gambling machines (EGMs), and online, and the number of hours and amount of money spent on gambling, to live alone, education, PHQ-2 and GAD-2 (≥ 3), time spent gambling online during the pandemic and the gambling frequency in the past 12 months were tested in the model.

### Analysis

For this study, descriptive analyses were conducted to describe the demographic characteristics of the sample. To address the first objective of this article, χ^2^ analysis was conducted, and alternatively, the Monte Carlo estimation was used when frequencies were deemed too small to compare gambling-related variables and seeking help variables across the four levels of the PGSI. To assess the second objective, Kruskal–Wallis tests (H) were conducted to analyse the continuous variables with abnormal distribution (e.g., money spent on gambling and number of gambling sessions). In addition, the categorical variables (PHQ-4 and gambling-related variables) were analysed by χ^2^. To respond to the third objective, the significant results of the last objective were used to perform a multivariate logistic regression, with the goal of identifying variables that could explain a PGSI score ≥ 3. In the final model, only variables with a *p* < 0.05 were conserved through stepwise selection. To alleviate the risk of error because of the many variables included in the analysis, a *p* < 0.05 was considered significant after using a false discovery rate correction. The analyses were conducted using SPSS V.28 software.

### Patient and public involvement

A patient partner who experienced gambling problems was involved in several stages of this study. We received feedback during the design of the research protocol and online questionnaire. She guided us in choosing the right words when discussing problematic gambling and the ideal length of an online questionnaire. We continue to seek feedback from patients and the public during the dissemination phase of our research findings.

## Results

### Profile of participants

A non-probabilistic sample of 973 gamblers completed the PGSI. As presented in table 1, most were employed (61.0%), White (93.1%) and females (51.9%). One quarter (24.7%) were non-risk gamblers (PGSI score = 0), 181 (18.6%) were low-risk gamblers (PGSI = 1–2), 271 (27.9%) were moderate-risk gamblers (PGSI = 3–7) and 281 (28.9%) of participants were high-risk gamblers (PGSI ≥ 8).

**Table 1:** Sociodemographic characteristics

Variables	n (%)
Gender (n = 973**)**	
Female	505 (51.9)
Male	461 (47.4)
Prefer not to answer	4 (0.4)
Other	3 (0.3)
Age (n = 973**)**	
18–24	71 (7.3)
25–34	221 (22.7)
35–44	267 (27.4)
45–54	190 (19.5)
55–64	167 (17.2)
65–74	53 (5.5)
75+	4 (0.4)
Group of belonging (n = 967**)**	
White	906 (93.7)
Black	12 (1.2)
Asian	6 (0.6)
Arabic	11 (1.1)
Latino	6 (0.6)
Native	21 (2.2)
Other	5 (0.5)
Region of residence (n = 973**)**	
Montréal	174 (17.9)
Montérégie	150 (15.4)
Capitale-Nationale	150 (15.4)
Estrie	85 (8.7)
Laurentides	64 (6.6)
Lanaudière	58 (6.0)
Other	292 (30.0)
Marital status (n = 958**)**	
Single	341 (35.6)
Common law spouse	375 (39.1)
Married	166 (17.3)
Separated, divorced	61 (6.4)
Widow	15 (1.6)
Occupation (n = 971**)**	
Employed	592 (61.0)
Self-employed	87 (9.0)
Student	82 (8.4)
Retired	104 (10.7)
Unemployed	66 (6.8)
Stopped for sickness or disabled	29 (3.0)
Other	11 (1.1)
Education (n = 967**)**	
No diploma	54 (5.6)
High school diploma	192 (19.9)
Trade school	158 (16.3)
College diploma	237 (24.5)
Certificate	88 (9.1)
Bachelor’s degree	166 (17.2)
University diploma superior to bachelor’s degree	72 (7.5)

### Profile of gambling habits

#### Participation in types of gambling

Regarding the first objective of this study, the results indicated statistically significant associations between PGSI scores and each of the following gambling behaviours in the last 12 months: playing lottery tickets, gambling in casinos, using slot machines or EGMs and gambling online. No-risk gamblers seemed more likely to play lotto than the other participants (table 2). While low-risk, moderate-risk and high-risk gamblers do not differ regarding gambling in casinos, the likelihood of gambling online is greater for moderate and high-risk gamblers than it is for low-risk gamblers. No significant difference was noted between gambling on scratch-off tickets, bingo, poker, sports betting, horse racing, stock exchange, other types of gambling and the PGSI score of the participants.

**Table 2:** Type of gambling according to the level of the gambling severity index

	Non-problem gamblers **n (%**)	Low-risk **n (%**)	Moderate-risk **n (%**)	Problem gamblers **n (%**)	χ2	*p* value
Types of gambling						
N	240	181	271	281		
Scratch-off tickets	135 (56.3)	117 (64.6)	155 (57.2)	145 (51.6)	7.67	0.053
Bingo	28 (11.7)	17 (9.4)	33 (12.2)	35 (12.5)	1.15	0.765
Poker	37 (15.4)	40 (22.1)	55 (20.3)	45 (16.0)	4.80	0.187
Sports betting	28 (11.7)	28 (15.5)	43 (15.9)	32 (11.4)	3.67	0.299
Horse racing	1 (0.4)	0 (0.0)	2 (0.7)	1 (0.4)	1.36	0.847*
Stock exchange	15 (6.3)	7 (3.9)	19 (7.0)	12 (4.3)	3.22	0.359
Lotto	207 (86.3)	133 (73.5)	205 (75.7)	208 (74.0)	14.64	0.002
Casinos	33 (13.8)	50 (27.6)	88 (32.5)	93 (33.1)	30.73	<0.001
Slot machines	87 (36.3)	91 (50.3)	171 (63.1)	207 (73.7)	81.51	<0.001
Electronic gambling machines	11 (4.6)	11 (6.1)	40 (14.8)	76 (27.1)	66.22	<0.001
Other	3 (1.3)	1 (0.6)	1 (0.4)	3 (1.1)	1.59	0.733*
Online gambling	164 (68.3)	156 (86.2)	254 (93.7)	260 (92.5)	84.10	<0.001
Having a gambling problem						
Yes	0 (0.0)	6 (3.3)	69 (26.3)	222 (80.1)	626.90	<0.001
No	238 (99.2)	157 (87.2)	128 (48.9)	19 (6.9)		
Don’t know	2 (0.8)	17 (9.4)	65 (24.8)	36 (13.0)		
N	240	180	262	277		
Having a gambling problem in the past						
Yes	6 (2.5)	17 (9.8)	46 (23.2)	17 (28.8)	115.22	<0.001*
No	231 (96.3)	146 (84.4)	129 (63.6)	27 (45.8)		
Don’t know	3 (1.3)	10 (5.8)	23 (11.6)	15 (25.4)		
N	240	173	198	59		
Having requested self-exclusion						
Yes	1 (16.7)	1 (4.4)	21 (18.3)	94 (39.8)	26.97	<0.001*
No	5 (83.3)	22 (95.7)	94 (81.7)	142 (60.2)		
N	6	23	115	236		
Having consulted resources for their gambling problem						
Yes	0 (0.0)	1 (4.4)	16 (14.0)	79 (33.2)	23.34	<0.001*
No	6 (100.0)	22 (95.7)	98 (86.0)	159 (66.8)		
N	6	23	114	238		

* Monte Carlo estimation.

#### Gambling problems, self-exclusion and resources

Respondents who reported having a gambling problem in the past were more likely to be high-risk gamblers, as per their PGSI scores (table 2). In the overall study sample, only 96 people consulted resources about gambling, including 79 (33.2%) high-risk gamblers, 16 (14%) moderate-risk gamblers and 1 (4.4%) low-risk gambler. Among the resources consulted, the most consulted were addiction workers (52.6%), helplines (46.3%), self-help groups (38.9%), psychologists (34.7%), entourage (33.6%) and family doctors (26.3%).

### Impacts of the COVID-19 pandemic on gamblers’ mental health and gambling habits

Regarding the second objective of this study, the results indicated that problem gamblers (PGSI ≥ 8) reported significantly more symptoms of anxiety and depression in the past 2 weeks than their low- and moderate-risk counterparts (table 3).

**Table 3:** Anxiety and depression scores for the past 2 weeks according to the level of the gambling severity index

	Non-problem gamblers (n=229)	Low-risk (n=175)	Moderate-risk (n=261)	Problem gamblers (n=270)	χ2	*p*value
PHQ-4						
≤ 2	132 (57.6)	73 (41.7)	96 (36.8)	54 (20.0)	122.53	<0.001
3–5	46 (20.1)	52 (29.7)	82 (31.4)	60 (22.2)		
6–8	33 (14.4)	25 (14.3)	43 (16.5)	56 (20.7)		
≥ 9	18 (7.9)	25 (14.3)	40 (15.3)	100 (37.0)		
PHQ-2 ≥ 3	46 (20.1)	55 (31.4)	99 (37.9)	171 (63.3)	105.15	<0.001
GAD-2 ≥ 3	51 (22.3)	51 (29.1)	74 (28.4)	147 (54.4)	69.23	<0.001

GAD-2, Generalised Anxiety Disorder-2; PHQ-2, Patient Health Questionnaire-2; PHQ-4, Patient Health Questionnaire-4.

The likelihood of reporting an increase in general gambling habits during the pandemic was greater for low-risk, moderate-risk and high-risk gamblers than it was for no-risk gamblers (table 4). Furthermore, 85.1% of high-risk gamblers reported an increase in their gambling habits during the pandemic, compared with 77.9% of the moderate-risk gamblers, 59.1% of the low-risk gamblers and 30.4% of the no-risk gamblers. Regarding online gambling, the same pattern of results was observed for both online state and private platforms. Regarding state platforms, moderate-risk and high-risk gamblers were more likely to report an increase in their online gambling. Regarding private platforms, high-risk gamblers were more likely to report an increase in their online gambling.

**Table 4:** Impacts of COVID-19 on gambling habits

	Non-problem gamblers	Low-risk	Moderate-risk	Problem gamblers	Statistic	*p* value
General gambling habits					χ2=235.56	<0.001
Decrease	26 (10.8)	17 (9.4)	29 (10.7)	22 (7.8)		
No impact	141 (58.8)	57 (31.5)	31 (11.4)	20 (7.1)		
Increase	73 (30.4)	107 (59.1)	211 (77.9)	239 (85.1)		
N	240	181	271	281		
Online gambling on state platforms					χ2=114.18	<0.001
Decrease	7 (4.4)	7 (4.8)	8 (3.4)	10 (4.1)		
No impact	90 (57.0)	53 (36.1)	36 (15.1)	37 (15.3)		
Increase	61 (38.6)	87 (59.2)	194 (81.5)	195 (80.6)		
N	158	147	238	242		
Online gambling on private platforms					χ2=72.27	<0.001*
Decrease	3 (4.0)	1 (1.1)	4 (3.0)	6 (3.3)		
No impact	48 (64.0)	42 (47.7)	42 (31.1)	27 (14.8)		
Increase	24 (32.0)	45 (51.1)	89 (65.9)	150 (82.0)		
N	75	88	135	183		
Test of new ways to gamble					χ2=185.76	<0.001
Yes	47 (19.6)	83 (45.9)	178 (65.7)	212 (75.4)		
No	193 (80.4)	98 (54.1)	93 (34.3)	69 (24.6)		
N	240	181	271	281		
Time available to gamble					χ2=143.82	<0.001
Decrease	9 (3.8)	10 (5.5)	17 (6.3)	17 (6.1)		
No impact	137 (57.6)	66 (36.5)	50 (18.5)	38 (13.5)		
Increase	92 (38.7)	105 (58.0)	204 (75.3)	226 (80.4)		
N	238	181	271	281		
Money available to gamble					χ2=143.36	<0.001
Decrease	19 (8.0)	14 (7.8)	48 (18.0)	82 (29.7)		
No impact	182 (76.8)	108 (60.3)	118 (44.2)	78 (28.3)		
Increase	36 (15.2)	57 (31.8)	101 (37.8)	116 (42.0)		
N	237	179	267	276		
Number of gambling sessions per year before the pandemic					H=53.81	<0.001
Median	24.5	48	52	60		
IQR 25 to 75	5 to 72	12 to 104	12 to 120	52 to 156		
N	240	181	269	279		
Number of gambling sessions per year during the pandemic					H=218.23	<0.001
Median	48	104	208	260		
IQR 25 to 75	10 to 104	48 to 208	96 to 312	156 to 364		
N	239	181	271	278		
Money spent per year on gambling before the pandemic					H=152.34	<0.001
Median	300	520	1300	3120		
IQR 25 to 75	56.25 to 1040	112 to 1370	480 to 4160	1040 to 10 400		
N	240	181	271	279		
Money spent per year on gambling during the pandemic					H=396.99	<0.001
Median	480	1040	4160	15 600		
IQR 25 to 75	100 to 1200	360 to 2800	1800 to 10 400	5200 to 31 200		
N	239	181	271	280		

Values were presented with n (%) except for continuous variables that were presented with median and IQR when indicated.

* Monte Carlo estimation.

As presented in table 4, moderate-risk and high-risk gamblers were more likely to report having tried new ways to gamble during the pandemic. Low-risk, moderate-risk and high-risk gamblers were more likely to report having more time to gamble during the pandemic compared with their no-risk gambler counterparts. In addition, while the money spent per year on gambling increased during the pandemic for every group, the higher the increase in the amount spent, the greater the PGSI score. While gambling frequency during the pandemic increased for every group compared with before the pandemic, the higher the increase in frequency, the greater the PGSI score.

### Problematic gambling during the pandemic

The third objective of this article was to identify, with a multivariate logistic regression, the factors that predisposed problematic gambling (moderate or high-risk gambling) during the COVID-19 pandemic. As shown in table 5, the final model is composed of 11 independent variables that explain 50.9% of the variance in problematic gambling (*R^2^* de Nagelkerke = 0.509). The results indicated that individuals experiencing changes in marital status or job loss, increased tobacco consumption or fluctuations in gambling expenditures due to COVID-19 were more likely to exhibit problematic gambling behaviours (ie, PGSI ≥ 3). Regarding the frequency of gambling during the past 12 months, it was observed that the greater the frequency, the greater the risk of having problematic gambling behaviours. In addition, people experiencing symptoms of depression (i.e., PHQ-2 ≥ 3), who gamble in casinos, with slot machines, with EGMs or online, are more likely to have problematic gambling behaviours. Furthermore, individuals with postsecondary education, those who reported decreased tobacco consumption due to COVID-19 and non-tobacco users were less likely to have a PGSI score of 3 or higher, therefore considered problematic gamblers.

**Table 5:** Stepwise multivariate logistic regression of factors that predispose problem gambling severity index ≥ 3 (n = 840)

	OR (95% CI)	*p* value
Impact of COVID-19 on marital status	3.39 (1.35 to 8.48)	0.009
Job loss due to COVID-19	2.00 (1.12 to 3.55)	0.019
Education		
High school diploma or less	1	
Trade school	0.76 (0.41 to 1.39)	0.371
College diploma	0.46 (0.27 to 0.77)	0.003
University diploma	0.57 (0.35 to 0.94)	0.027
Impact of COVID-19 on tobacco consumption		
No impact	1	
Decrease	0.67 (0.26 to 1.71)	0.400
Increase	1.89 (1.06 to 3.37)	0.032
Does not apply	0.74 (0.49 to 1.11)	0.145
PHQ-2 ≥ 3	2.13 (1.44 to 3.17)	<0.001
Playing in casino	1.61 (1.05 to 2.46)	0.028
Playing with slot machines	2.34 (1.59 to 3.46)	<0.001
Playing with electronic gambling machines	3.28 (1.80 to 5.98)	<0.001
Online gambling	2.33 (1.19 to 4.56)	0.014
Impact of COVID-19 on gambling expenditures		
No impact	1	
Decrease	6.96 (3.49 to 13.86)	<0.001
Increase	5.53 (3.48 to 8.79)	<0.001
Frequency of gambling for the past 12 months		
A few times during the year	1	
A few times by month	2.42 (1.19 to 4.9)	0.015
A few times by week	4.69 (2.33 to 9.47)	<0.001
Almost every day	10.31 (4.57 to 23.29)	<0.001
Every day	15.35 (5.39 to 43.75)	<0.001

R2(Nagelkerke) = 0.509.

PHQ-2, Patient Health Questionnaire-2.

## Discussion

This study aimed to examine how the COVID-19 pandemic affected gambling behaviours and mental health among adult gamblers in Quebec. The first objective was to describe gambling habits and problem gambling among gamblers. Overall, our results show that participants with a higher PGSI score are more likely to play slot machines and EGMs, to report having a gambling problem in the present or in the past, to have requested self-exclusion and to have consulted resources for gambling problems than the participants with a lower PGSI score. However, participants with a lower PGSI score are more likely to play lotto than the others. In addition, the results show that the likelihood of having increased gambling habits during the pandemic is greater for low-risk, moderate-risk and problem gamblers than no-risk gamblers.

The second objective was to explore the general impacts of the pandemic on the mental health and gambling habits of gamblers. The results suggest that the pandemic impacted gamblers’ mental health and their gambling habits. The positive associations found between the PGSI scores and the symptoms of depression and anxiety could be explained by social isolation and stress linked to the pandemic,^27^ as well as gambling online.^62^ Moreover, while a high proportion of our sample reported having a gambling problem, either currently or in the past, only a few sought counselling, resources or self-exclusion help.

In their narrative review about the help-seeking behaviours of problem gamblers, Loy *et al.*^63^ found that many studies observed that only 5%–20% of problem gamblers sought help. This could be explained by many factors, including shame, denial of the problem and/or of the need to seek help and various social factors.^64 65^ It is worth considering that the COVID-19 context may have influenced individuals’ ability or inclination to seek counselling help or use self-exclusion measures. Factors such as limited access to services, concerns about health risks or self-reliance due to lockdowns might have affected help-seeking behaviours for mental health.^66^ However, the way of studying help-seeking behaviours varies considerably among studies and can therefore affect the disparity in the results.^67^ For example, in our study, help-seeking behaviour was studied in the lifetime of the participants and included any type of help-seeking, compared with some studies that restrict help-seeking behaviours to professional help and/or to a limited period. Indeed, in our study, 35.9% of the high-risk gamblers who reported having sought help for their gambling behaviours had sought help from someone in their entourage, which could explain why 33.2% of the high-risk gamblers in our study had reported seeking help in their lifetime.

Regarding the impacts of the pandemic on participants’ gambling habits, most of the participants reported an increase in their online gambling, their time available to gamble and in their frequency of gambling. The increase in online gambling during the pandemic observed in our sample is coherent with what has been reported by many studies, as shown by the literature reviews of Hodgins and Stevens,^68^ Sachdeva *et al.*^69^ and Catalano *et al*.^70^ This does not seem surprising, given the imposed public health measures limiting the possibility of gambling at land-based venues. According to an epidemiological point of view, accessibility is an essential factor for engaging in a behaviour.^71^ Therefore, the limitation of the possibility of going to land-based gambling venues can be interpreted as an important change in the environment that limits access to physical gambling possibilities. However, people still having access to online gambling possibilities could then explain the increase in online gambling during the pandemic. Overall, an increased gambling frequency during the pandemic can be explained by gamblers’ boredom and their common perception that gambling could help cope with anxiety and depression.^68^ Furthermore, the pandemic seems to have impacted the money available to problem gamblers to gamble, who report an increase in their money available, compared with the other groups of gamblers who reported no impact of the pandemic on their money available to gamble.^46 62 72 73^ In their original research, Gainsbury *et al.*^74^ had the objective of studying the impacts of gambling venue shutdowns in Australia. Contrary to our study, they found a decrease in general gambling and in online gambling during the pandemic among their participants, with a small subset of participants reporting an increase in their general and online gambling habits during the pandemic. Despite the fact that not knowing the disparity of PGSI scores among their sample, it is possible to hypothesise that the difference in the results could be explained by a higher proportion of moderate-risk and problem gamblers in our sample. The third objective was to identify the factors that predisposed people to problematic gambling behaviours during the pandemic. Regarding the type of gambling, gambling in casinos, with slot machines, with EGMs and online predicted problematic gambling in our sample. These results are in line with other studies in which an association between gambling with EGMs and problematic gambling was observed.^75^,^77^ Regarding psychosocial factors, a change in marital status, job loss and the presence of symptoms of depression were found to be the predictors of problematic gambling in the sample. Finally, increased tobacco use, increased and decreased gambling expenditures and high gambling frequency in the last 12 months predicted problematic gambling.

### Strengths and limitations

This study presents an overview of the impacts of the COVID-19 pandemic on 973 gamblers in Quebec. In particular, given the broad range of PGSI scores in the sample, distinguishing the impacts of the pandemic on low-risk, moderate-risk and high-risk gamblers was possible.

While using social media and organisation websites for recruitment provided a wide reach, this non-random sampling may have introduced selection bias, as it primarily targeted individuals who are active online and have easy access to online platforms, have already engaged with gambling-related organisations or have sought help for their gambling problem. This could explain why the proportion of problem gamblers is higher than that typically observed in other studies,^47 78 79^ which affects the representativeness of the sample and limits the generalisability of the findings. Nonetheless, given the proportion of problem gamblers in the sample, providing an overview of the factors that put individuals at risk for problematic gambling during the pandemic was possible. It is also estimated that this study can therefore give an overview of the impact of the pandemic on people with gambling problems, which could be insightful in case of a future pandemic. Although the use of the short versions of the mental health scales limited the interpretation of participants’ mental states, the goal of this study was to gain insights into an overview of the overall impacts of the COVID-19 pandemic on people who gamble, rather than its specific impacts on anxiety and depression. Furthermore, this research provides an overview of the impact of the first year of the pandemic, not the entire pandemic period; therefore, it is important to contextualise the results. Finally, while the questionnaire covered a wide range of relevant topics, the length (85 items) might have contributed to participant fatigue and reduced response rates.

## Conclusions

This study highlights the significant impact of the COVID-19 pandemic on gambling behaviours in Quebec and identifies the factors associated with problematic gambling. Moving forward, it is crucial to prioritise mental health support for people who gamble, particularly in post-pandemic recovery initiatives. Efforts should focus on developing accessible resources and interventions to address the reluctance to seek help among people indulging in problematic gambling practices, as was highlighted in our study. In addition, proactive measures are needed to prevent and mitigate the negative effects of future pandemics on gambling behaviours. By fostering collaboration among researchers, policymakers and stakeholders, we can work toward creating a safer and more supportive environment for individuals affected by problematic gambling, both during and after the pandemic. The next phase of this study, which aims to delve deeper into the qualitative experiences of people who gambled during the pandemic, will be presented in an upcoming article.

## Data Availability

Data are available upon reasonable request.
