# Supplementary material for: Gambling and the COVID-19 pandemic in the province of Quebec (Canada): results from an online cross-sectional survey of people who had gambled within the last 12 months
Source: BMJ Open. 2026 Mar 26;16(3):e097944. doi: 10.1136/bmjopen-2024-097944 (PMC13034363; doi:10.1136/bmjopen-2024-097944)
Supplement: Supplementary file 1 [file bmjopen-2024-097944.reviewer_comments.pdf]

PEER REVIEW HISTORY

BMJ Open publishes all reviews undertaken for accepted manuscripts. Reviewers are asked to complete a checklist review form and are provided with free text boxes to elaborate on their assessment. These free text comments are reproduced below.

ARTICLE DETAILS

Title (Provisional)

Gambling and the COVID-19 pandemic in the province of Quebec (Canada): results from an online cross-sectional survey of people who had gambled within the last 12 months

Authors

Brodeur, Magaly; Fortier, Marie-Ève; Carrier, Nathalie; Audette-Chapdelaine, Sophie; Auger, Anne-Marie; Savard, Annie-Claude; Kairouz, Sylvia

---

VERSION 1 - REVIEW

**Reviewer**	**1**
**Name**	**Battersby, Malcolm**
**Affiliation**	**Flinders University, Psychiatry**
**Date**	**04-Apr-2025**
**COI**	**None**

The COVID pandemic provided a unique opportunity or natural experiment to see how citizens responded to social disruption and the closure of land-based gambling in terms of their gambling behaviour. This Canadian study is aiming to describe gambling behaviour and its consequences in 973 gamblers in Quebec. This is a descriptive quantitative study surveyed people who responded to social media advertising between Feb and March 2021.

It is not a representative or random sample of the general population. The main eligibility criteria is having gambled at least once in the last 12 months. The results showed that 56% were moderate to severe risk gamblers which included 28.9% who were high risk gamblers. This immediately indicates that this was a skewed or highly selected group of gamblers. One comparison is that in most developed countries the proportion of the whole population with high risk gambling is in the range of 1-5%. Not surprisingly the high risk gamblers scored more highly on depression and anxiety than moderate or low risk gamblers.

Hence I think the title is slightly misleading and could state – ‘results from an on-line cross sectional survey of people who had gambled within the last 12 months’.

In terms of help seeking table 2 raw numbers are presented but in the text this is broken down by individual resources eg addiction workers (55.13%). Can this be clarified which % this is – what % in each group/risk category sought help and I presume that it is of those who sought help the percentages apply?

The items in table 4 ask the person to estimate before and after pandemic levels of number of sessions, money spent etc. In money spent, all groups are higher during than during the pandemic however only high risk gamblers 'reported an increase in their gambling budget' – does this just mean that they spent more or was it statistically significant and the other groups weren't as from the table there are large increases in most of the groups? This is confusing which the authors could clarify.

The discussion confirms that at risk gamblers were more likely to increase their gambling during the pandemic than no risk gamblers. This is an unsurprising finding.

Confirms other research that in addition to high risk, low and moderate risk gamblers have negative consequences from their gambling and that the concept of 'risk' is misleading in that in other conditions eg anxiety and depression symptom scales are used to define low, moderate and severe conditions ie the person is not 'at risk' but actually has negative symptoms and consequences. The terminology of 'risk' also conveys to politicians, the public and industry that the gambling behaviour and consequences isn't a serious issue as it's 'a risk' ie the problem hasn't actually occurred yet.

There is little that is new or surprising in these results but it adds to the general literature that gambling and its consequence did not disappear or reduce during COVID but in fact worsened in some jurisdictions but a similar study in Australia had the opposite finding of reduced gambling https://pmc.ncbi.nlm.nih.gov/articles/PMC8562923/. To add to the interest and value of the study the authors could comment on possible explanations for this difference in these 2 studies.

A general comment about the longitudinal aspect of the study ie pre and during the pandemic is that it confirms that gambling disorder is a psychiatric condition which can be chronic and relapsing and remitting like any other addiction and does not disappear when one means of gambling is removed.

In terms of help seeking the overall % of help seeking should be repeated to put in context the data from other studies cited.

In comparing their results with other studies three systematic reviews of gambling during the pandemic are cited. There is no commentary of how this study's results compare with this literature with a particular focus on the sampling of only at risk gamblers. Similarly for predictors of problematic gambling the authors cite 3 studies but are not specific about psychosocial studies as comparitors to their own results.

In limitations the authors note the selection bias based on on-line users limiting the generalisability of the findings.

I could not see where ethics approval was recorded or if respondents received a gift voucher?

**Reviewer**	**2**
**Name**	**Seangpraw, Katekaew**
**Affiliation**	**University of Phayao, School of Public health**
**Date**	**17-Oct-2025**
**COI**	**None**

1. This research is interesting, but due to the incomplete data collection and the long duration of data gathering, the information or circumstances may have changed. Therefore, the research design should be adjusted, such as adopting a cohort or retrospective or prospective study.

2. The introduction section lacks a connection to the concept of self-exclusion. It should be expanded to highlight and clarify the research problem.

3. Regarding the sampling technique, it is important to clearly explain how the participants were recruited in order to minimize potential bias. Since the data collection was conducted through online questionnaires, there is a risk of selection bias—particularly the overrepresentation of individuals who are more technologically literate, have internet access, or are more motivated to respond. This method may inadvertently exclude certain demographics, such as older adults, those with limited digital access, or individuals less engaged with online platforms. Therefore, the sampling strategy and its limitations should be explicitly addressed to enhance the credibility and generalizability of the research findings.

4. The discussion section should be strengthened by integrating relevant theoretical frameworks that underpin the study's findings. This will not only provide academic grounding but also demonstrate how the results align with or challenge existing theories. Additionally, the discussion should explore the practical implications of the findings, particularly in terms of policy development. Highlighting how the research can inform public policy, guide strategic interventions, or support evidence-based decision-making will enhance the study's relevance and societal impact.

VERSION 1 - AUTHOR RESPONSE

[R1] The COVID pandemic provided a unique opportunity or natural experiment to see how citizens responded to social disruption and the closure of land-based gambling in terms of their gambling behaviour. This Canadian study is aiming to describe gambling behaviour and its consequences in 973 gamblers in Quebec. This is a descriptive quantitative study surveyed people who responded to social media advertising between Feb and March 2021. It is not a

representative or random sample of the general population. The main eligibility criteria is having gambled at least once in the last 12 months. The results showed that 56% were moderate to severe risk gamblers which included 28.9% who were high risk gamblers. This immediately indicates that this was a skewed or highly selected group of gamblers. One comparison is that in most developed countries the proportion of the whole population with high risk gambling is in the range of 1-5%. Not surprisingly the high risk gamblers scored more highly on depression and anxiety than moderate or low risk gamblers. Hence I think the title is slightly misleading and could state – 'results from an on-line cross sectional survey of people who had gambled within the last 12 months'.

We appreciate your suggestion and the explanation of the reasoning behind it. We changed the title as you suggested.

[R1] In terms of help seeking table 2 raw numbers are presented but in the text this is broken down by individual resources eg addiction workers (55.13%). Can this be clarified which % this is – what % in each group/risk category sought help and I presume that it is of those who sought help the percentages apply?

Thank you for your comment. We understand that the information available in Table 2 is limited for fully understanding the search for help. However, few subjects reported seeking help, so we have little information about the professionals consulted. We combined the percentages for the entire sample who reported consulting professionals about their gambling problem. We have added information to the text to clarify which group/risk category sought help.

[R1] The items in table 4 ask the person to estimate before and after pandemic levels of number of sessions, money spent etc. In money spent, all groups are higher during than during the pandemic however only high risk gamblers 'reported an increase in their gambling budget' – does this just mean that they spent more or was it statistically significant and the other groups weren't as from the table there are large increases in most of the groups? This is confusing which the authors could clarify.

Thank you for your comment. It wasn't statistically significant. To avoid confusion, we have removed it.

[R1] The discussion confirms that at risk gamblers were more likely to increase their gambling during the pandemic than no risk gamblers. This is an unsurprising finding. Confirms other research that in addition to high risk, low and moderate risk gamblers have negative consequences from their gambling and that the concept of 'risk' is misleading in that in other conditions eg anxiety and depression symptom scales are used to define low, moderate and severe conditions ie the person is not 'at risk' but actually has negative symptoms and consequences. The terminology of 'risk' also conveys to politicians, the public and industry that the gambling behaviour and consequences isn't a serious issue as it's 'a risk' ie the problem hasn't actually occurred yet.

As presented in our Methods' section, we used the Problem Gambling Severity Index (PGSI)

to assess problem gambling. This instrument uses the term “risk” when categorizing gambling subtypes, and is often used in the literature. Hence, we decided to stay coherent to the instrument subtypes and the literature.

[R1] There is little that is new or surprising in these results but it adds to the general literature that gambling and its consequence did not disappear or reduce during COVID but in fact worsened in some jurisdictions but a similar study in Australia had the opposite finding of reduced gambling https://pmc.ncbi.nlm.nih.gov/articles/PMC8562923/. To add to the interest and value of the study the authors could comment on possible explanations for this difference in these 2 studies.

The results of the study and an explanation for the disparity in the results has been provided in our “Discussion” section.

[R1] A general comment about the longitudinal aspect of the study ie pre and during the pandemic is that it confirms that gambling disorder is a psychiatric condition which can be chronic and relapsing and remitting like any other addiction and does not disappear when one means of gambling is removed. In terms of help seeking the overall % of help seeking should be repeated to put in context the data from other studies cited.

Thank you for your comment. We have added information about this in the discussion section.

[R1] In comparing their results with other studies three systematic reviews of gambling during the pandemic are cited. There is no commentary of how this study’s results compare with this literature with a particular focus on the sampling of only at risk gamblers. Similarly for predictors of problematic gambling the authors cite 3 studies but are not specific about psychosocial studies as comparitors to their own results. In limitations the authors note the selection bias based on on-line users limiting the generalisability of the findings.

Thank you for your comment. We have added a few lines to the discussion section to explain how our study relates to the existing literature.

[R1] I could not see where ethics approval was recorded or if respondents received a gift voucher?

As mentioned in the ethical approval statement at the end of the manuscript, this study was approved by the Scientific and Research Ethics Committee of the Centre intégré universitaire de santé et de services sociaux de l'Estrie — Centre hospitalier universitaire de Sherbrooke (CIUSSS de l'Estrie-CHUS) under number #2021-3818. We have added the following statement to the ethical statement: As part of this research project, participants received no compensation for their participation in the online questionnaire.

[R2] This research is interesting, but due to the incomplete data collection and the long duration of data gathering, the information or circumstances may have changed. Therefore,

the research design should be adjusted, such as adopting a cohort or retrospective or prospective study.

Our data was collected for four weeks, between February 16th and March 15th in 2021 (4 weeks), which represents a standard data gathering for this type of study. We therefore consider that modifying the research design is not necessary.

[R2] The introduction section lacks a connection to the concept of self-exclusion. It should be expanded to highlight and clarify the research problem.

The main objective of our study was to examine the impact of the COVID-19 pandemic on adult gamblers' behaviors and mental health. Even though we asked the participants about the behavior of having used self-exclusion in the past, this concept was not the central point in our study. We therefore consider it not necessary to add a section about this concept in our introduction section.

[R2] Regarding the sampling technique, it is important to clearly explain how the participants were recruited in order to minimize potential bias. Since the data collection was conducted through online questionnaires, there is a risk of selection bias—particularly the overrepresentation of individuals who are more technologically literate, have internet access, or are more motivated to respond. This method may inadvertently exclude certain demographics, such as older adults, those with limited digital access, or individuals less engaged with online platforms. Therefore, the sampling strategy and its limitations should be explicitly addressed to enhance the credibility and generalizability of the research findings.

We agree with the bias that an online questionnaire can create, which was stated in our "Strength and limitations" section. We added clarification about the impact of the online questionnaire as you proposed.

[R2] The discussion section should be strengthened by integrating relevant theoretical frameworks that underpin the study's findings. This will not only provide academic grounding but also demonstrate how the results align with or challenge existing theories. Additionally, the discussion should explore the practical implications of the findings, particularly in terms of policy development. Highlighting how the research can inform public policy, guide strategic interventions, or support evidence-based decision-making will enhance the study's relevance and societal impact.

Thank you for your comment. We have added a discussion about accessibility and the environment in the discussion section. In the conclusion section, we address the policies that could be adopted in the post-pandemic period.
